# Glycerol Biosynthesis Pathways from Starch Endow *Dunaliella salina* with the Adaptability to Osmotic and Oxidative Effects Caused by Salinity

**DOI:** 10.3390/ijms26147019

**Published:** 2025-07-21

**Authors:** Huiying Yao, Yi Xu, Huahao Yang, Yihan Guo, Pengrui Jiao, Dongyou Xiang, Hui Xu, Yi Cao

**Affiliations:** Microbiology and Metabolic Engineering Key Laboratory of Sichuan Province, Key Laboratory of Bio-Resources and Eco-Environment of Ministry of Education, College of Life Sciences, Sichuan University, Chengdu 610065, China

**Keywords:** *Dunaliella salina*, salinity stress, glycerol metabolism, starch metabolism, glycerol-3-phosphate shuttle

## Abstract

*Dunaliella salina*, a unicellular and eukaryotic alga, has been found to be one of the most salt-tolerant eukaryotes with a wide range of practical applications. To elucidate the underlying molecular mechanisms of *D. salina* in response to salinity stress, we performed transcriptome sequencing on samples under different stress conditions. A total of 82,333 unigenes were generated, 4720, 1111 and 2611 differentially expressed genes (DEGs) were identified under high salt stress, oxidative stress and hypertonic stress, respectively. Our analysis revealed that *D. salina* responds to salinity stress through a complex network of molecular mechanisms. Under high salt stress, starch degradation is regulated by AMY (α-amylase) and PYG (glycogen phosphorylase) with alternative expression patterns. This process is hypothesized to be initially constrained by low ATP levels due to impaired photosynthesis. The clustering analysis of DEGs indicated that starch and sucrose metabolism, as well as glycerol metabolism, are specifically reprogrammed under high salt stress. Glycerol metabolism, particularly involving GPDHs, plays a crucial role in maintaining osmotic balance under salinity stress. Key glycerol metabolism genes were up-regulated under salinity conditions, indicating the importance of this pathway in osmotic regulation. The G3P shuttle, involving mitochondrial GPDHs (c25199_g1 and c23777_g1), contributes to redox imbalance management under high salt, oxidative and hypertonic stresses. Notably, c23777_g1 is involved in the G3P shuttle under high salt, oxidative and hypertonic stresses, while c25199_g1 is specifically induced by hypertonic stress. The R2R3-MYB gene (c23845_g1) may respond to different effects of salinity stress by regulating the transcription of ROS-related genes. Our study provides a detailed understanding of the molecular responses of *D. salina* to salinity stress. We reveal the critical roles of starch and sucrose metabolism, glycerol metabolism and transcription factors in the *D. salina* adaptation to salinity.

## 1. Introduction

*Dunaliella salina*, a halophilic, unicellular, eukaryotic alga, is one of the most salt-tolerant eukaryotic organisms to date. It can survive at NaCl concentrations ranging from 0.05 mol/L to 5.5 mol/L [1]. Compared with other halophilic plants, *D. salina* cells have the advantages of a short growth period, high fecundity and no cell wall [2], making them a model species for studying plant adaptation to salt stress. At present, the salinity stress response of *D. salina* has been studied in terms of physiology, biochemistry, transcriptional regulation and proteomics [1,3,4,5,6].

Osmotic stress is one of the main factors affecting plant growth in high salt environments. The synthesis of soluble organic small molecules in the cytoplasm, including betaine [7,8], proline [7,9], *myo*-inositol [10], mannitol [11] and sorbitol [12], can reduce the osmotic potential of cells under salt stress. Glycerol, a single compatible solute, plays a dominant role in the osmotic regulation of *D. salina* [13]. *D. salina* cells can maintain intracellular osmotic pressure by synthesizing and accumulating large amounts of glycerol under high salt stress, and the content of glycerol can reach up to 50% of the dry weight of the algae under NaCl saturation conditions [14]. The DHAP produced by the glycolytic pathway and the Calvin cycle pathway is catalyzed by GPDH to produce glycerol 3-phosphate, which is then catalyzed by GPP to produce glycerol [15]. Starch, a common carbohydrate, was found to be one of the raw materials for glycerol biosynthesis induced by salt stress under light [16]. Hypertonic stress can be simulated by the addition of NaCl or sorbitol in algal culture, and some common effects have been found in previous studies. For example, Takashi Yuasa et al. found activation of 40 kDa protein kinases in response to hyperosmotic shock treated by NaCl or sorbitol in *Dunaliella tertiolecta* [17]. However, a study on the distinctions and relationships between NaCl and sorbitol treatments in *D. salina* is not yet available, and whether a single sorbitol treatment will lead to changes in metabolic processes such as starch hydrolysis and biosynthesis of osmolytes is also unknown.

Salinity stress is accompanied by a burst of reactive oxygen species (ROS), which could result in oxidative damage [18,19,20]. Agal species have developed defiance systems to detoxify and eliminate stress-induced ROS including antioxidants such as carotenoids, ascorbic acid and glutathione and antioxidant enzymes such as superoxide dismutase (SOD), catalase (CAT), peroxidase (POD), ascorbate peroxidase (APX), glutathione peroxidase (GPX) and glutathione S-transferase (GST) [21,22,23,24]. It was reported that the H_2_O_2_ content and O^−2^ generation rate of *D. salina* were increased and declined, respectively, at 6 h after salinity shock. Meanwhile, the activities of SOD and GPX were increased, whereas the activities of enzymes (i.e., APX, POD, CAT and GST) were declined [25]. Similarly, the activities of SOD, CAT and GPX had a significant increase in *D. tertiolecta* on exposure to an extreme range of salinity [26]. In addition, it has been reported that exogenous H_2_O_2_ and the osmotic stress induced by PEG significantly increased the activities of antioxidant enzymes such as SOD, CAT, GPX, APX and GR in the leaves of two cucumber cultivars, Jinchun no. 4 and Lvfeng no. 6 [27]. Oxidative stress occurs when *D. salina* is exposed to adverse environmental conditions such as high salt and H_2_O_2_. However, the relationship between salinity stress and H_2_O_2_-induced oxidative effects is not yet fully studied. Physiology and biochemistry responses to salinity stresses are controlled by regulating the expression of functional genes involved in different metabolic processes (i.e., glycerol synthesis [28], starch degradation [15] and ROS metabolism [29]) and genes encoding regulatory proteins such as transcription factors (TFs) [30]. Under salt stress, starch, glycerol and antioxidant activity work together to enhance the salt tolerance of the *Dunaliella* species. Starch provides a carbon source, glycerol acts as an osmotic regulator and antioxidant and the antioxidant system elevates the activities of protective enzymes to detoxify and eliminate ROS [26]. As one of the most important regulators, the roles of TFs in stress responses have attracted the attention of researchers and abundant TFs, which mainly belong to MYB, AP2/ERF, WRKY, NAC and bZIP families, have been identified and characterized as involved in stress responses in the last few decades [31,32]. Diverse TFs may respond to the same stress. For example, the transgenic *Arabidopsis* and *Tobacco* with over-expressing *TaWRKY10*, *TaWRKY79* or *TaWRKY93* exhibited enhanced tolerance to salinity stresses [33,34,35]. In addition, a transcription factor may function in diverse stress responses. For example, *TaODORANT1*, an R2R3-MYB gene from wheat (*Triticum aestivum* L.), was up-regulated under several treatments such as NaCl, PEG6000, ABA and H_2_O_2_ [36]. The transgenic tobacco with over-expressing *TaODORANT1* exhibited enhanced tolerance to both drought and salt stresses by up-regulating the expression levels of several ROS- and stress-related genes [36]. However, there is limited research on the functions of transcription factors in *D. salina*, and it is currently unclear whether transcription factors are involved in coordinating salt stress.

Salinity stress causes osmotic stress and oxidative stress, but there is almost no exploration of the different effects of salinity stress and salinity on *D. salina* at the whole transcriptional level. Therefore, to illuminate the underlying molecular mechanisms involved in the response to salinity and its different effects, we performed transcriptome sequencing on *D. salina* samples under different stresses, including high salt stress and other abiotic stresses (hypertonic stress and oxidative stress), which are associated with salinity stress. RT-qPCR was used to evaluate the reliability of transcriptome data. Differentially expressed genes (DEGs) were identified by comparisons between the control and experimental groups, and some representative DEGs were compared among the different stress samples to elucidate the distinct genes and pathways responding to abiotic stresses in *D. salina*. Therefore, in order to elucidate the potential molecular mechanisms of salt algae’s response to salinity and its different effects, we performed transcriptome sequencing on salt algae cells under different stresses, including high salt stress and other abiotic stresses related to salinity stress (high osmotic stress and oxidative stress). This study advances the understanding of the molecular responses to salinity and its diverse effects in *D. salina*.

## 2. Results

### 2.1. Sequencing, Reads Assembly, Gene Annotation and Functional Analysis

Twelve cDNA libraries were constructed and sequenced from algae cells with different stresses (Table S1). After the Illumina (San Diego, CA, USA) sequencing, approximately 603.85 million raw reads were obtained, and the average rate of clean reads was 84.17% (Table S1). After removal of low-quality data, all high-quality reads were assembled into 218,608 contigs and 126,222 transcripts using the de novo assembly program Trinity (v. r20140717), with N50 values of 497 bp and 1337 bp, respectively. A total of 82,333 unigenes with a mean length of 631.17 bp, a maximum length of 17,240 bp and an N50 value of 1036 bp were obtained. The results of the sequence assembly are presented in Table S2. About 69,060 (83.89%), 8529 (10.36%), 3046 (3.70%) and 1683 (2.05%) unigenes ranged from 100 bp to 999 bp, 1000 bp to 1999 bp, 2000 bp to 2999 bp and >3000 bp in length, respectively (Figure S1).

All assembled unigene sequences were matched against NR, GO, KEGG, eggNOG and Swiss-Prot. According to the BLAST (v2.7) results, a total of 17,917 (21.76%), 4533 (5.51%), 3446 (4.19%), 15,408 (18.71%) and 20,070 (24.38%) unigenes were matched in the Nr, GO, KEGG, eggNOG and Swiss-Prot databases. The result of the BLAST search analysis showed that a total of 4158 (23%), 3423 (19%) and 1282 (7%) unigenes in the NR annotations were matched with the sequences from *Volvox carteri f. nagariensis*, *Chlamydomonas reinhardtii* and *Coccomyxa subellipsoidea* C-169 (Figure S2). According to GO annotation, most of the unigenes were assigned to cellular component (CC) (40%), biological processes (BP) (38.5%) and molecular function (MF) (21.5%) (Figure S3). The biochemical pathways were predicted by the KEGG database, and a total of 3446 (4.19%) unigenes were assigned into 34 pathways (Figure S4).

### 2.2. Identification of Differentially Expressed Genes (DEGs)

**Figure 1:**
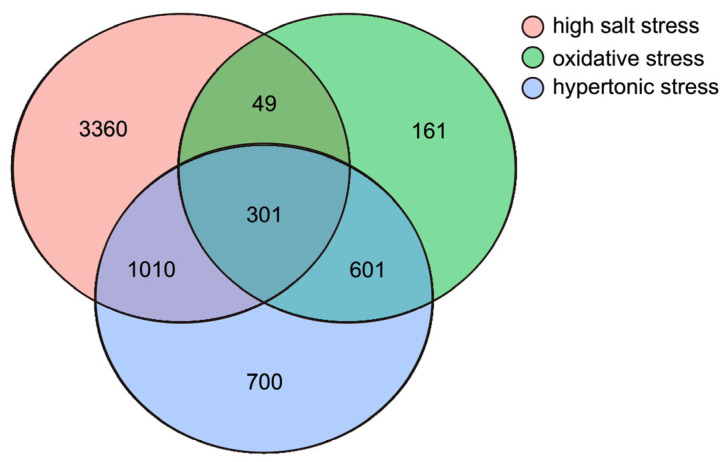
Venn diagram of all differentially expressed genes derived from different treatments.

**Figure 2:**
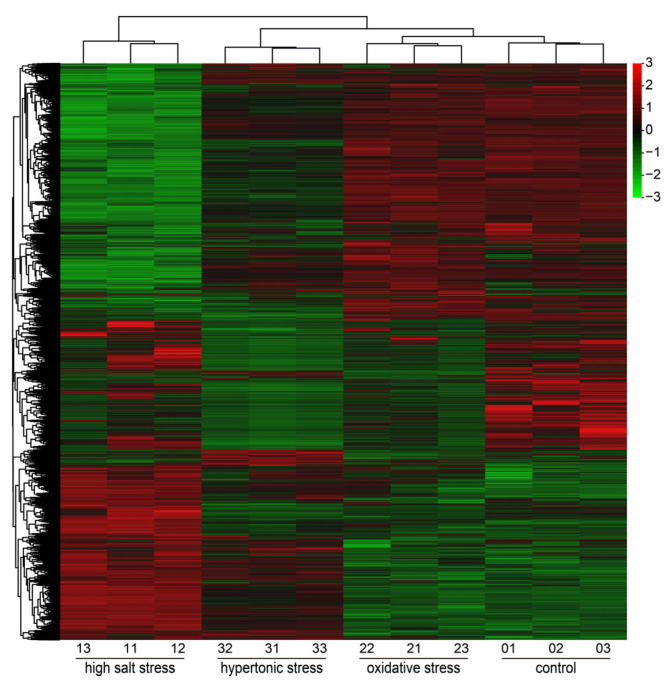
Cluster analysis of all DEGs derived from the control, high salt, oxidative and hypertonic stresses. Each treatment consists of three biological repeats: 01, 02, 03 (control); 11, 12, 13 (high salt stress); 21, 22, 23 (oxidative stress); and 31, 32, 33 (hypertonic stress).

Each stress sample was compared with the control group to identify the DEGs (log_2_ (fold change) > 1, *p*-value < 0.05). The highest number of DEGs was identified in the high salt-stress sample, with 4720 (1756 up- and 2964 down-regulated), 1111 DEGs (100 up- and 1011 down-regulated) were identified in the oxidative stress sample and 2611 DEGs (383 up- and 2228 down-regulated) were detected in the hypertonic stress sample (Figure 1 and Figure 2 and Table S3).

### 2.3. RNA-Seq Expression Validation by RT-qPCR

**Figure 3:**
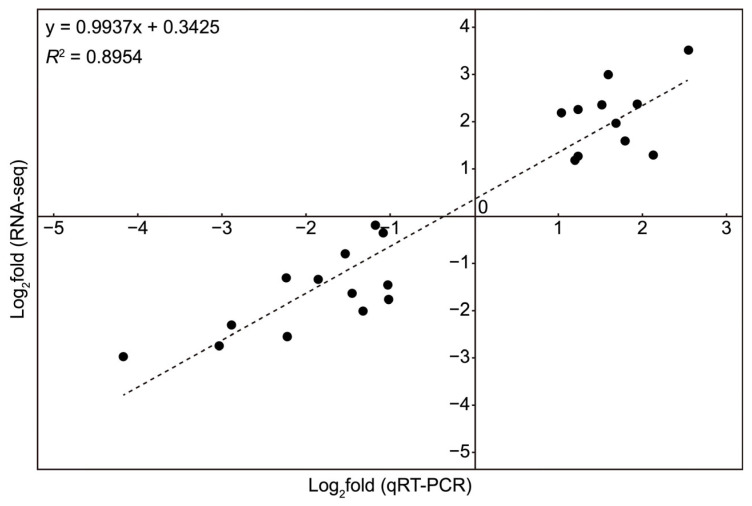
Correlation analysis between RNA-Seq and quantitative RT-qPCR results for 24 randomly selected DEGs (8 DEGs from high salt stress, 8 from oxidative stress and 8 from hypertonic stress).

To verify the reliability of the transcriptome data, a total of 24 DEGs were randomly selected for real-time quantitative PCR (RT-qPCR) analysis, including 8 DEGs derived from high salt stress, 8 DEGs derived from oxidative stress and 8 DEGs derived from hypertonic stress. As expected, the RNA-Seq and RT-qPCR results showed a significant correlation (*r*^2^ = 0.8954, n = 24, Figure 3), indicating that the DEGs identified by RNA-seq are reliable.

### 2.4. Glycerol Metabolism Pathway in Response to Salinity and Hypertonic Stresses

**Figure 4:**
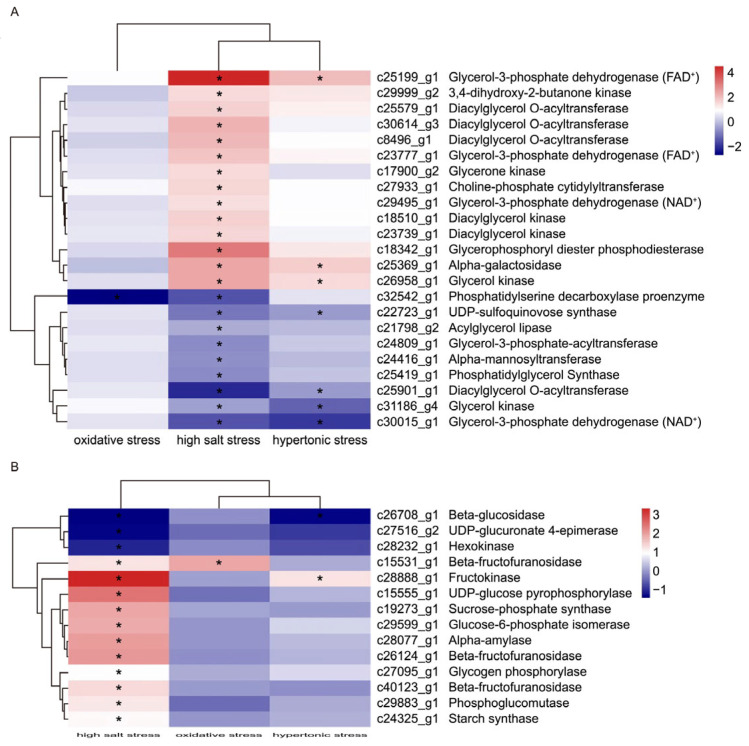
Heat map of DEGs involved in glycerol metabolism (**A**) and starch and sucrose metabolism (**B**) derived from different stresses. Navy and firebrick3 colors indicate down- and up-regulated transcripts, respectively. DEGs (|log2 (fold change) | > 1 and *p*-value < 0.05)) under different stresses were indicated by “*”.

Glycerol biosynthesis is an important mechanism for *D. salina* to cope with salt stress. To investigate the differences in glycerol metabolism of *D. salina* under different stress treatments, we conducted a cluster analysis based on DEGs (Figure 4A). Among them, the highest number of DEGs involved in glycerol metabolism was identified under high salt stress, with 23 DEGs (14 up- and 9 down-regulated); meanwhile, under oxidative and hypertonic stress, 1 DEG (down-regulated) and 7 DEGs (3 up- and 4 down-regulated) were identified, respectively (Figure 4A). As expected, most DEGs related to glycerol metabolism were up-regulated under salinity conditions, indicating that this pathway plays an important role in osmotic regulation. GPDH was identified as a key enzyme of glycerol synthesis in *D. salina* [37]. In this study, three up-regulated *GPDHs* were identified during salinity stress, including c29495_g1, c25199_g1 and c23777_g1, and the expression levels of these *GPDHs* increased by 2.2, 28.5 and 3.1-fold, respectively. Sorbitol treatment causes a hypertonic stress, which is somewhat like salinity treatment. Interestingly, *GPDH* (c25199_g1), a gene with the highest fold change (28.5-fold) among the differentially expressed *GPDHs* under salinity stress, was also increased 3.5-fold during hypertonic stress. But other differentially expressed *GPDHs* (c29495_g1 and c23777_g1) were not significantly affected by hypertonic stress. Moreover, three genes (c8496_g1, c25579_g1 and c30614_g3) encoding diacylglycerol O-acyltransferase and two genes (c23739_g1 and c18510_g1) encoding diacylglycerol kinase were up-regulated under salinity stress. However, the expression levels of these five genes were not significantly affected by oxidative and hypertonic stresses. In addition, several genes involved in glycerol metabolism, including a glycerol-3-phosphate dehydrogenase [NAD (+)] (c30015_g1) and a glycerol kinase (c31186_g4) were down-regulated both during salinity and hypertonic stresses. These results indicated that glycerol metabolism in response to hypertonic stress occurred in a more moderate way compared with salinity stress, but it was not induced by oxidative stress.

### 2.5. Starch and Sucrose Metabolism in Response to Salinity Stress

The products derived from photosynthesis and starch breakdown may be responsible for glycerol formation [38]. Therefore, we investigated the differences in starch and sucrose metabolism among different stresses. Approximately 14 DEGs involved in starch and sucrose metabolism were detected in comparisons between stress samples and the control. Among the 14 DEGs, 11 were up-regulated under salinity stress, including *PYG* (glycogen phosphorylase; c27095_g1) and *AMY* (alpha-amylase; c28077_g1), but only one gene (c15551_g1 and c28888_g1) was up-regulated under oxidative stress and hypertonic stress, respectively (Figure 4B). As expected, the expression levels of most DEGs involved in starch and sucrose metabolism under salt stress were different from the other groups (Figure 4B). These results indicated that starch and sucrose metabolism were reprogrammed under high salt-stress conditions.

### 2.6. The KEGG Enrichment of Salinity-Responsive Pathways Involved in Starch Degradation

**Figure 5:**
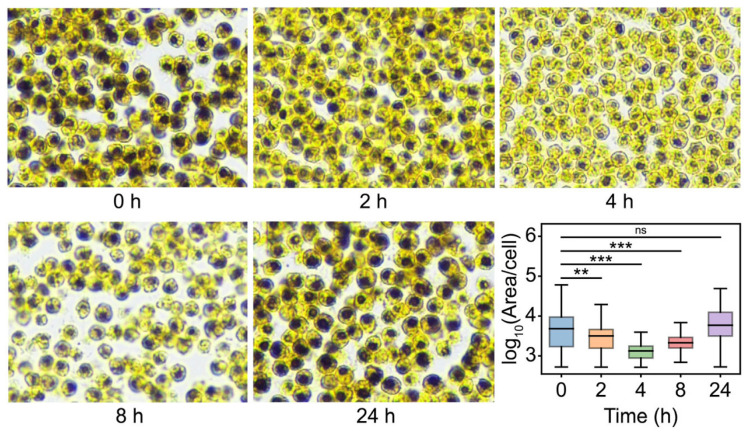
Iodine-starch staining experiment. Samples were collected at 0 h, 2 h, 4 h, 8 h and 24 h after salinity shock from a medium containing 3 M NaCl. Starch granules are stained blue or blue–black. Box plots reflecting the area of intracellular starch-stained regions of *D. salina* at different time points. A two-sided *t*-test was conducted on samples from the experimental group compared to the 0 h time point to assess significant differences. The results indicated significant differences as follows: ** *p*  <  0.01, *** *p*  <  0.001 and ns (not significant).

**Figure 6:**
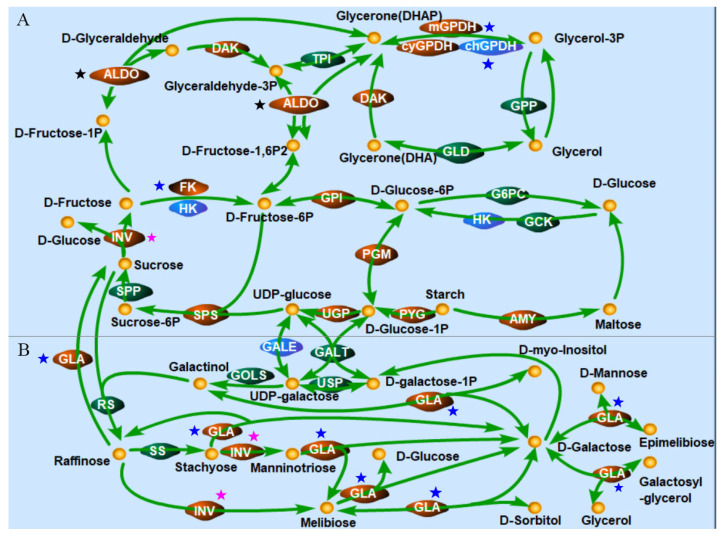
The KEGG enrichment of salinity-responsive pathways involved in starch degradation. Starch and sucrose metabolism, fructose and mannose metabolism, glycolysis and glycerol synthetic pathway (**A**), and galactose metabolism (**B**). Red, blue and green colors indicate up-regulated, down-regulated and no change transcripts, respectively. Black stars indicate common transcripts among high salt, hypertonic and oxidative stresses; blue stars indicate common transcripts between high salt and hypertonic stresses and pink stars indicate common transcripts between high salt and oxidative stresses. AMY: alpha-amylase [EC:3.2.1.1]; PYG: glycogen phosphorylase [EC:2.4.1.1]; UGP: UTP-glucose-1-phosphate uridylyltransferase [EC:2.7.7.9]; SPS: sucrose-phosphate synthase [EC:2.4.1.14]; SPP: sucrose-6-phosphatase [EC:3.1.3.24]; INV: beta-fructofuranosidase [EC:3.2.1.26]; FK: fructokinase [EC:2.7.1.4]; HK: hexokinase [EC:2.7.1.1]; GPI: glucose-6-phosphate isomerase [EC:5.3.1.9]; PGM: phosphoglucomutase [EC:5.4.2.2]; G6PC: glucose-6-phosphatase [EC:3.1.3.9]; GCK: glucokinase [EC:2.7.1.2]; GALE: UDP-glucose 4-epimerase [EC:5.1.3.2]; GALT: UDPglucose-hexose-1-phosphate uridylyltransferase [EC:2.7.7.12]; USP: UDP-sugar pyrophosphorylase [EC:2.7.7.64]; GOLS: inositol 3-alpha-galactosyltransferase [EC:2.4.1.123]; RS: raffinose synthase [EC:2.4.1.82]; SS: stachyose synthetase [EC:2.4.1.67]; GLA: alpha-galactosidase [EC:3.2.1.22]; ALDO: fructose-bisphosphate aldolase, class I [EC:4.1.2.13]; DAK: triose/dihydroxyacetone kinase/FAD-AMP lyase (cyclizing) [EC:2.7.1.282.7.1.29 4.6.1.15]; mGPDH: glycerol-3-phosphate dehydrogenase [EC:1.1.5.3]; cyGPDH, chGPDH: glycerol-3-phosphate dehydrogenase (NAD+) [EC:1.1.1.8]; GPP: glycerol 3-phosphatase [EC:3.1.3.21]; TPI: triosephosphate isomerase [EC:5.3.1.1]; GLD: glycerol dehydrogenase [EC:1.1.1.6].

As expected, the content of starch was gradually reduced from 0 h to 4 h after salinity shock, whereas the starch content started rising at 8 h and returned to the primary level at 24 h after salinity shock (Figure 5). These results indicated that the degradation of starch may be an important mechanism of *D. salina* in response to salt stress. To elucidate this mechanism, the KEGG enrichment analysis of salinity-responsive pathways involved in starch degradation was performed (Figure 6). The products of starch breakdown are mainly used for the biosynthesis of glycerol through the starch and sucrose metabolism, fructose and mannose metabolism and glycolysis and glycerol synthetic pathway (Figure 6A). In the starch and sucrose metabolism pathway, the expression level of genes encoding eight enzymes (AMY, PYG, UGP, PGM, GPI, SPS, INV and FK) was increased, generating three different pathways for starch hydrolysis (Figure 6A). In addition, UDP-glucose released during starch breakdown feeds into the galactose metabolism pathway (Figure 6B). In this pathway, genes encoding GLA (alpha-galactosidase [EC:3.2.1.22]) and INV (β-Fructofuranosidase [EC:3.2.1.26] were up-regulated, and as a result, many osmoprotectants were synthesized, such as melibiose, D-sorbitol, D-myo-inositol and D-mannose (Figure 6B). It is noteworthy that the number of DEGs involved in starch degradation under hypertonic and oxidative stresses was far less than salinity stress, despite several common transcripts being observed among the different treatments.

### 2.7. The Dynamic Expression of Key Genes Involved in Starch Hydrolysis and Glycerol Biosynthesis

**Figure 7:**
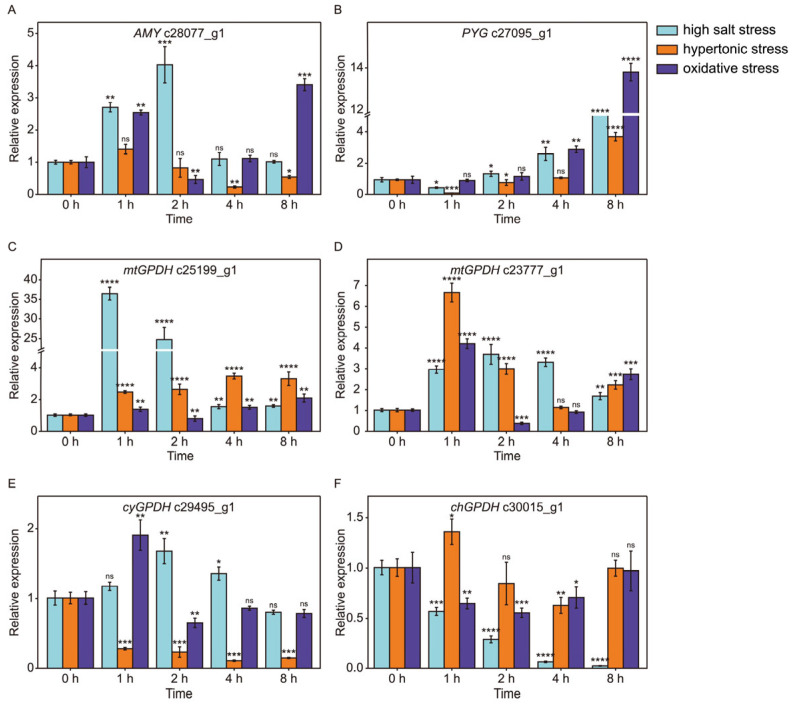
The dynamic expression of key genes involved in starch hydrolysis and glycerol biosynthesis. AMY (**A**), PYG (**B**), mtGPDH (**C**,**D**), cyGPDH (**E**) and chGPDH (**F**). RT-qPCR was performed in triplicate. Light cyan, orange and violet colors represent high salt stress, hypertonic stress and oxidative stress, respectively. A two-sided *t*-test was conducted on samples from the experimental group compared to the 0 h time point to assess significant differences. The results indicated significant differences as follows: * *p*  <  0.05, ** *p*  <  0.01, *** *p*  <  0.001, **** *p*  <  0.0001 and ns (not significant).

The key enzymes AMY [EC:3.2.1.1] and PYG [EC:2.4.1.1] are related to the decomposition of starch in response to high salt shock (Figure 7A,B), but the expression patterns of genes encoding these two enzymes were different. The gene c28077_g1 encoding AMY responded quickly but briefly to salt stress. The expression level of c28077_g1 was about three times as much as the control group at 1 h after salinity shock and reached a peak value at 2 h and then returned to normal. Moreover, the c28077_g1 was also induced by oxidative stress but not by hypertonic stress (Figure 7A). In reverse, the gene c27095_g1 encoding PYG responded slowly but persistently to salt stress. The gene c27095_g1 was down-regulated at 1 h, and then the expression level of c27095_g1 increased gradually and reached 7.5-fold at 8 h under salinity stress. Meanwhile, we observed that the c27095_g1 was also induced by both hypertonic and oxidative stress, but it seems to be more sensitive to oxidative stress (Figure 7B). These results suggested that AMY and PYG functioned in the different stages of starch degradation and played different roles in salinity, causing oxidative and hypertonic effects. The glycerol synthetic pathway was particularly interesting because of the diversity of GPDH (Figure 6A). To understand the possible function of differentially expressed GPDHs, dynamic expression detection of these genes was performed (Figure 7C–F). Both genes c25199_g1 and c23777_g1 encode mitochondrial GPDHs (also known as FAD-GPDHs), but some differences were observed between them. The gene c25199_g1 was dramatically up-regulated at 1 and 2 h (36.5-fold and 24.5-fold, respectively) during salinity stress and returned to basal level at 4 h and 8 h. The expression of gene c25199_g1 was also mildly induced by hypertonic stress. However, it was not induced by oxidative stress during the whole process (Figure 7C). For gene c23777_g1, it was induced by all treatments (Figure 7D). The gene c29495_g1 encoding a cytoplasmic GPDH (NAD-GPDH) was positively related to salinity and oxidative stresses but inversely related to hypertonic stress (Figure 7E). In addition, the gene c30015_g1 encoding a chloroplastic GPDH seems to be down-regulated at salt shock only (Figure 7F). These results indicated that the glycerol-3-phosphate shuttle, which was performed by mitochondrial and cytoplasmic GPDHs to maintain redox homeostasis, might be an important mechanism for *D. salina* in response to environmental stresses.

### 2.8. The Dynamic Change in Some Antioxidant Enzyme Genes Involved in ROS Scavenging

**Figure 8:**
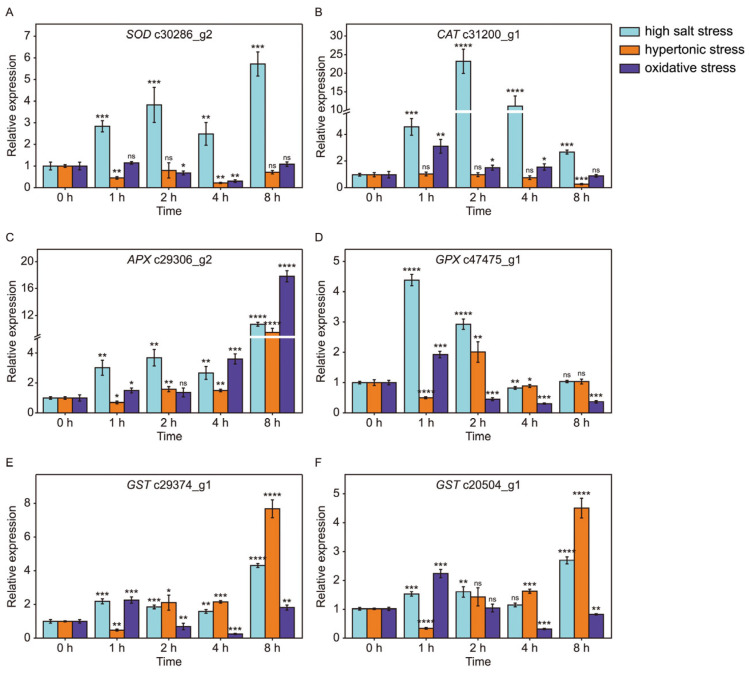
The dynamic expression of antioxidant enzyme genes. SOD (**A**), CAT (**B**), APX (**C**), GPX (**D**) and GST (**E**,**F**) RT-qPCR was performed in triplicate. Light cyan, orange and violet colors represent high salt stress, hypertonic stress and oxidative stress, respectively. A two-sided *t*-test was conducted on samples from the experimental group compared to the 0 h time point to assess significant differences. The results indicated significant differences as follows: * *p*  <  0.05, ** *p*  <  0.01, *** *p*  <  0.001, **** *p*  <  0.0001 and ns (not significant).

A cellular redox imbalance under abiotic stresses results in the production of ROS, and stress-induced ROS accumulation is mainly eliminated by various antioxidant enzymes. This study investigated the difference in antioxidant enzymes among salinity and its hypertonic effect and oxidative effect simulated by sorbitol and H_2_O_2_ treatment, respectively. The expression of several important antioxidant enzyme genes was monitored by RT-qPCR (Figure 8). Overtly, several antioxidant enzyme genes were induced by all stress treatments, including a gene encoding APX, a gene encoding GPX and two genes encoding GST (Figure 8C–F). In addition, the gene c30286_g2 encoding SOD was only induced by high salt stress but not by hypertonic and oxidative stresses (Figure 8A), and the gene c31200_g1 encoding CAT was induced by both high salt stress and oxidative stress, but not by hypertonic stress (Figure 8B). These results indicate that ROS scavenging by antioxidant enzymes is a common mechanism employed by *D. salina* to cope with diverse abiotic stresses, although some stress-specific antioxidant enzymes also exist.

### 2.9. Transcription Factors (TFs) Responding to Abiotic Stresses

**Figure 9:**
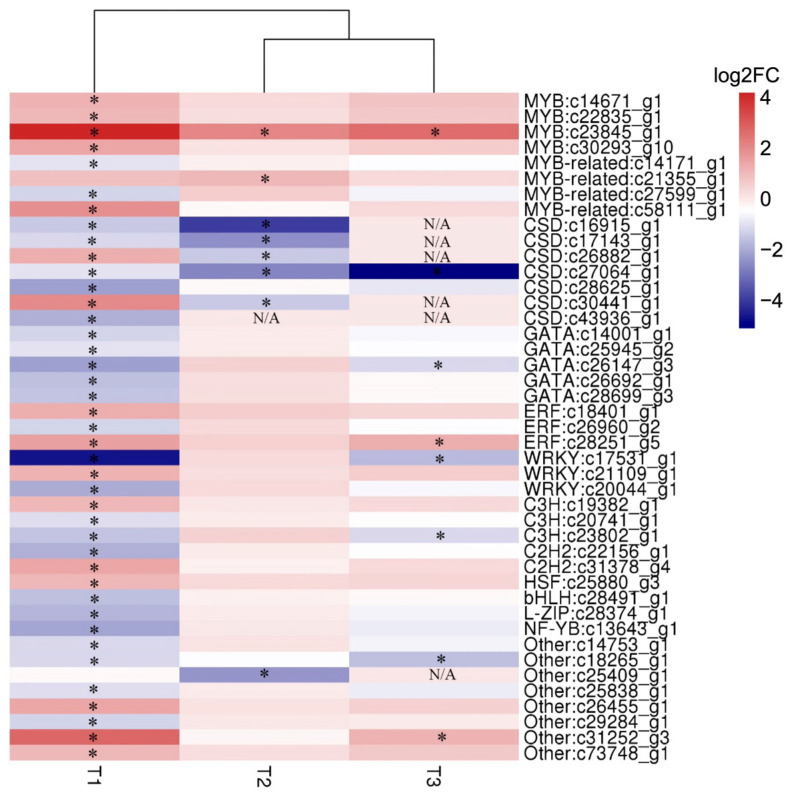
Heat map of differentially expressed transcription factors derived from different stresses. T1: high salt stress; T2: oxidative stress and T3: hypertonic stress. Navy and firebrick3 colors indicate down- and up-regulated transcripts, respectively. DEGs under different stresses were indicated by “*”. N/A: not applicable.

Transcription factors are important regulatory proteins that play irreplaceable roles in stress responses. To investigate the relationship at the level of transcription factors among salinity stress and its hypertonic effect and oxidative effect, the DEGs derived from different stresses encoding TFs were picked out to carry out cluster analysis (Figure 9). In total, 43 DEGs encoding TFs were identified, and these TFs belonged to 13 families, including MYB, MYB-related, CSD, GATA, ERF, WRKY and other families. The CSD family was the largest TF family responding to abiotic stresses and most DEGs encoding CSD were down-regulated under abiotic stresses. Similarly, the GATA family responding to salinity stress was typically down-regulated. Conversely, the MYB family responding to salinity stress was typically up-regulated. Moreover, the MYB-related, ERF, WRKY and C3H TF families also showed responses to salt stress, which include at least three DEGs. Notably, a gene (c23845_g1) encoding MYB transcription factor was pronouncedly up-regulated under all treatments, and the fold changes were 18.1, 4.1 and 6.2 under high salt stress, oxidative stress and hypertonic stress, respectively.

### 2.10. The Dynamic Expression of TFs Under Abiotic Stresses

**Figure 10:**
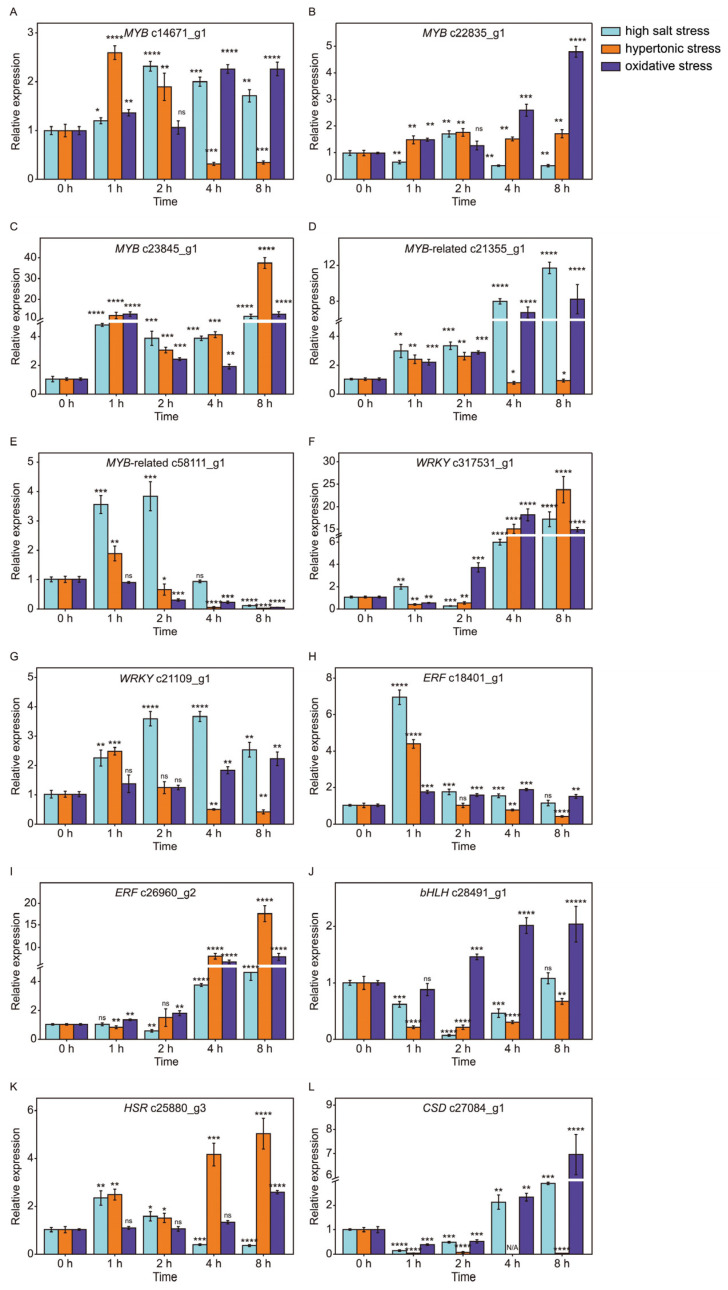
The dynamic expression of TFs under abiotic stresses. MYB (**A**–**E**), WRKY (**F**,**G**), ERF (**H**,**I**), bHLH (**J**), HSR (**K**) and CSD (**L**). RT-qPCR was performed in triplicate for 12 transcription factors belonging to 6 families: MYB family (c14671_g1, c22835_g1, c23845_g1, c21355_g1, c58111_g1), WRKY family (c17531_g1, c21109_g1), ERF family (c18401_g1, c26960_g2), bHLH family (c28491_g1), HSF family (c25880_g3) and CSD family (c27064_g1). Light cyan, orange and violet represent high salt stress, hypertonic stress and oxidative stress, respectively. A two-sided *t*-test was conducted on samples from the experimental group compared to the 0 h time point to assess significant differences. The results indicated significant differences as follows: * *p*  <  0.05, ** *p*  <  0.01, *** *p*  <  0.001, **** *p*  <  0.0001 and ns (not significant).

To further understand the possible functions of TFs in stress tolerance, twelve transcription factors were selected to perform time-scale gene expression analysis by RT-qPCR (Figure 10). For the MYB family, all three of them responded to salinity stress and oxidative stress, but the expression of gene c22835_g1 was not affected by hypertonic stress. Interestingly, the expression pattern of c23845_g1 was significantly different from c14671_g1 and c22835_g1 in consideration of its high fold change and similar expression patterns in response to all abiotic stresses (Figure 10A–C). For the MYB-related family, c21355_g1 showed the same expression patterns when exposed to salinity and oxidative stresses (Figure 10D). However, c58111_g1 showed reversed expression patterns under salinity and oxidative stresses (Figure 10E). The gene c17531_g1 encoding WRKY and c26960_g2 encoding ERF were visibly up-regulated in the latter stages of abiotic stresses (Figure 9). Nevertheless, c18401_g1 encoding ERF was up-regulated in the early stages of salinity and hypertonic stresses (Figure 10H). In addition, the gene c27064_g1 encoding CSD was distinctly down-regulated at hypertonic stress during the whole process, but it was up-regulated at 4 h and 8 h under both high salt and hypertonic stresses (Figure 10L). Notably, the gene c23845_g1 (MYB), which was observed up-regulated under all treatments in transcriptome data (Figure 9) was up-regulated during all stress treatments and its expression patterns were similar among high salt stress, oxidative stress and hypertonic stress (Figure 10C). The TFs had different expression patterns when *D. salina* was exposed to different abiotic stresses, which indicated that salinity stress and its hypertonic effect and oxidative effect might be regulated by different TFs and an MYB encoded by the gene c23845_g1 might be a common TF in response to different stress effects caused by salinity.

## 3. Discussion

### 3.1. Starch and Sucrose Metabolism as Well as Glycerol Metabolism Were Reprogrammed Under High Salt Stress Condition

Multiple metabolic pathways involved in starch hydrolysis and glycerol biosynthesis were changed at high salinity shock, including starch and sucrose metabolism, glycerol metabolism, fructose and mannose metabolism and glycolysis and galactose metabolic pathway (Figure 4 and Figure 6). Among these pathways, the starch and sucrose metabolism pathway involved in starch being degraded to D-fructose-6P showed significant reprogramming. A cascade of enzymes including eight enzymes ([EC:3.2.1.1], [EC:2.4.1.1], [EC:2.7.7.9], [EC:2.4.1.14], [EC:3.2.1.26], [EC:2.7.1.4], [EC:5.3.1.9] and [EC:5.4.2.2]) and one enzyme ([EC:2.7.1.1]) displayed positively and negatively correlated transcriptional profiles with salinities. However, only one enzyme ([EC:3.2.1.26] and [EC:2.7.1.4]) showed an elevated transcriptional profile with oxidative stress and osmotic stress, respectively (Figure 6). In addition, glycerol metabolism was evidently reprogrammed (Figure 4A). About 14 enzymes showed elevated transcriptional profile with salinity stress, while several (or no) enzymes displayed a positively correlated transcriptional profile with oxidative stress and osmotic stress. These results indicated that the reprogramming of pathways involved in starch hydrolysis and glycerol biosynthesis is caused by comprehensive effects but not a single effect, such as the oxidative effect and the osmotic effect of salinity stress.

### 3.2. Starch Serves as a Temporary Pioneer in Response to Salinity Stress Through Different Catabolic Pathways

Starch is a common carbohydrate which can be promptly decomposed to provide soluble sugars when plants are exposed to environmental stresses. Soluble sugars are the main osmolytes of many non-halophytes, and they can protect cytoplasmic membranes and enzymes from stress damage [39]. Studies have shown that there is an accumulation of soluble sugars and a depletion of starch content in leaves under salt and drought stresses [39,40,41]. Some previous studies have observed a depletion of starch content in *D. salina* after salt shock [15,28,38] and it was consistent with our study (Figure 5) The phenomenon of the starch content starting to rise at 8 h and returning to primary level at 24 h after salinity shock (Figure 5) indicated that starch served as a “temporary pioneer” to promptly alleviate salinity stress, but afterward the starch was re-synthesized within a short time period. Fang et al. proposed a viewpoint that photosynthetic sugar was a preferential carbon source for starch accumulation but not glycerol synthesis [28], which was consistent with the phenomenon we observed and supported our inference that starch served as a “temporary pioneer” at salt shock. RNA-seq analysis of *D. salina* in response to reciprocal salinity changes showed that in the starch hydrolysis pathway, four enzymes (EC 2.4.1.1, EC 5.3.1.9, EC 2.7.1.11 and EC 2.7.7.9) and five enzymes (EC 2.4.1.18, EC 3.1.3.11, EC 2.4.1.21, EC 2.7.7.27 and EC 5.4.2.2) displayed a positively and negatively correlated transcriptional profile with salinities [28]. In this study, most DEGs involved in starch and sucrose metabolism were significantly up-regulated under salinity stress. The enzyme AMY [EC:3.2.1.1] related to the decomposition of starch was also up-regulated in high salt conditions, besides the enzyme PYG [EC:2.4.1.1]. Moreover, the expression level of many genes related to the biosynthesis of D-fructose-6P were increased including phosphoglucomutase (PGM) [EC:5.4.2.2], glucose-6-phosphate isomerase (GPI) [EC:5.3.1.9], UTP-glucose-1-phosphate uridylyltransferase (UGP) [EC:2.7.7.9], sucrose-phosphate synthase (SPS) [EC:2.4.1.14], β-Fructofuranosidase (INV) [EC:3.2.1.26] and fructokinase (FK) [EC:2.7.1.4] (Figure 6A). These results indicated that in the early stages of salt stress, three pathways are responsible for starch transforming into D-fructose-6P (1: Starch—D-glucose-1P—D-glucose-6P—D-fructose-6P, 2: Starch—D-glucose-1P—UDP-glucose—Sucrose-6P—Sucrose—D-fructose—D-fructose-6P and 3: Starch—Maltose—D-glucose—D-glucose-6P—D-fructose-6P) rather than a single pathway (Starch—D-glucose-1P—D-glucose-6P—D-fructose-6P) which had been reported in previous studies [28,42]. We propose that multiple pathways might be beneficial to improve the efficiency of starch degradation during the initial stages of salt stress and produce more abundant soluble sugars, which can also act as osmolytes besides glycerol.

### 3.3. Starch Breakdown Might Be Triggered Mainly by Salinity Causing Oxidative Effect and Regulated by Both AMY and PYG with Alternative Expression Patterns Under Salt Stress

A depletion of starch content in *D. salina* at salinity stress has been observed in many studies [15,28]. Chitlaru et al. [42] deemed that PYG [EC:2.4.1.1] was responsible for starch degradation to synthesize glycerol. Fang et al. found that in the starch hydrolysis pathway, the gene encoding PYG was up-regulated at 4 h after salinity changes from 0.5 M to 2 M [28], and it is consistent with our result (Figure 7B). In our study, the enzyme AMY [EC:3.2.1.1] related to the decomposition of starch was also raised in salt stress besides the enzyme PYG [EC:2.4.1.1] (Figure 6A). The dynamic expression of genes encoding AMY and PYG show that AMY responded quickly but briefly to salt stress, whereas PYG responded slowly but persistently to salt stress (Figure 7A,B). Therefore, we propose that AMY is a major contributor to catalyze starch degradation at the beginning of salt stress (0–2 h after salinity shock) and PYG is a dominating enzyme for starch hydrolysis in a long-term salt stress response. Starch hydrolysis by AMY is a spontaneous process and does not cause a depletion of ATP, while PYG catalyzing starch hydrolysis is driven by ATP. Therefore, we propose that PYG activity is constrained at the onset of salt stress by a dwindling ATP supply resulting from impaired photosynthesis and its rapid diversion to immediate defense reactions; however, over the long term, PYG-driven starch hydrolysis becomes central because it is the quickest way to generate abundant metabolic intermediates that sustain salinity acclimation. Salinity stress causes diversified effects, including an osmotic effect and oxidative effect, which were mentioned by the previous research [43,44,45]. But research on salinity causing an osmotic effect and oxidative effect in the starch hydrolysis pathway has not so far been studied deeply. In this study, the gene encoding AMY was induced by both salinity and oxidative stress but not by hypertonic stress (Figure 7A). The gene encoding PYG is induced by both hypertonic and oxidative stress, but it seems to be more sensitive to oxidative stress (Figure 7B). Therefore, we propose that starch breakdown at high salt shock might be triggered mainly by salinity, causing an oxidative effect rather than an osmotic effect.

### 3.4. Glycerol-3-Phosphate Shuttle and Antioxidant Enzymes Participate in Solving the Problem of Redox Imbalance Under Abiotic Stresses

Glycerol biosynthesis affects the ratio of NADH/NAD^+^ during abiotic stresses. The maintenance of the redox balance of cells by modulating the cytosolic NADH/NAD^+^ ratio is a necessity for sustained cellular metabolism. Glycerol-3-phosphate (G3P) shuttle is one of the important redox adjustment mechanisms which has been extensively studied in yeast, animal systems and *Arabidopsis* [46,47,48]. In the G3P shuttle, dihydroxyacetone phosphate (DHAP) is converted to G3P by cytosolic GPDH with a consumption of NADH, and the generated G3P passes through the permeable outer membrane of mitochondria. G3P is re-oxidized to DHAP by FAD-dependent glycerol 3-phosphate dehydrogenase (FAD-GPDH), which is located on the outer surface of the mitochondrial inner membrane and simultaneously delivers electrons to the respiratory chain. Subsequently, DHAP returns to the cytoplasm. Thus, the G3P shuttle channels cytosolic reducing equivalents to mitochondria without utilizing a metabolite transporter. Previous studies have shown that G3P, the substrate of FAD-GPDH, is an integral precursor for the biosynthesis of glycerol. In addition, it is well known that the biosynthesis of glycerol is a vital mechanism of *D. salina* in response to high salt shock. Previously, our laboratory successfully isolated a FAD-GPDH (the ID in the transcriptome is c23777_g1) from *D. salina* [49]. In this study, two differentially expressed mitochondrial GPDHs (FAD-GPDHs) were found in *D. salina* under abiotic stresses (Figure 4A and Figure 7C,D). One of the mtGPDH (c23777_g1) was positively correlated with all abiotic stresses (Figure 7D) and the other mtGPDH (c25199_g1) was induced by NaCl significantly and sorbitol mildly, respectively (Figure 7C). In addition, a cytosolic GPDH (c29495_g1) showed a positive correlation with high salt and oxidative stresses but a negative correlation with osmotic stress (Figure 7E). These results suggest that the G3P shuttle might also exist in *D. salina* and might be an important mechanism for *D. salina* to adapt to suboptimal environmental stresses. We proposed that the c23777_g1 is a common gene of *D. salina* involved in G3P shuttle under high salt, oxidative and hypertonic stresses and the c25199_g1 is a unique gene of G3P shuttle induced by hypertonic stress. A cellular redox imbalance under abiotic stresses results in the production of ROS. Reactive oxygen species (ROS) such as O_2_^−^, H_2_O_2_ and HO· hinder the normal growth, development and metabolism of organisms because of their oxidative damage to lipids, nucleic acids and proteins [50]. Environmental stresses such as salinity, drought and extreme temperature are always accompanied by the burst-out of ROS [20,51,52]. To balance the ROS of cells, organisms have evolved a set of enzymes and a non-enzymatic antioxidant defense system. Stress-induced ROS accumulation is mainly eliminated by various antioxidant enzymes such as superoxide dismutase (SOD), catalase (CAT), peroxidase (POD), ascorbate peroxidase (APX), glutathione peroxidase (GPX) and glutathione S-transferase (GST) [53,54,55,56]. In addition, antioxidants such as glutathione (GSH), ascorbic acid (AsA) and procyanidins also play an important role in the removal of reactive oxygen species [54,57,58]. In this study, we found that the expression of genes encoding GST, GPX and APX, which are involved in the GSH-AsA cycle, were induced by all treatments (Figure 8C–F), and this will inevitably lead to increased consumption of GSH and AsA along with the removal of H_2_O_2_. Furthermore, one gene encoding SOD, a unique enzyme involved in dismutation of superoxide into H_2_O_2_ and oxygen, increased at high salt shock only (Figure 8A). These results indicate that different abiotic stresses may produce different reactive oxygen species and induce the gene expression of different antioxidant enzymes, but ROS scavenging by antioxidant enzymes is a common mechanism of *D. salina* to deal with different abiotic stresses.

### 3.5. A R2R3-MYB Gene (c23845_g1) Might Be a Core TF in Response to Different Stresses by Regulating the Transcription of ROS-Related Genes

*TaODORANT1*, an R2R3-MYB gene from wheat (*Triticum aestivum* L.), was up-regulating under several treatments such as NaCl, PEG6000, ABA and H_2_O_2_ [36]. The transgenic tobacco with over-expressing *TaODORANT1* exhibited an enhanced tolerance to both drought and salt stresses by up-regulating the expression levels of several ROS- and stress-related genes [36]. Similarly, BnaMYB78, an R2R3-MYB gene from canola (*Brassica napus* L.), regulates the transcription of a few ROS- and defense-related genes [59]. In addition, Zhu et al. found that an R2R3-type MYB gene OsMYB91 has a function in salt stress tolerance in rice. OsMYB91 over-expression plants showed higher levels of ROS scavenging enzymes and a lower content of H_2_O_2_ and MDA at salt stress [60]. In this study, we observed that a gene (c23845_g1) encoding MYB transcription factor was pronouncedly up-regulated and showed similar expression patterns under high salt, oxidative and hypertonic stresses (Figure 9 and Figure 10C). In addition, multiple antioxidant enzyme genes were induced by all stress treatments, including APX, GPX and GST (Figure 8C–F). Combining the previous research and our results, we propose that an MYB gene (c23845_g1) might be a common TF in response to different stresses by regulating the transcription of ROS-related genes.

## 4. Materials and Methods

### 4.1. Materials and Stress Treatments

*Dunaliella salina* (strain 435), obtained from the Institute of Hydrobiology, Chinese Academy of Sciences, was grown in defined medium [61] under controlled conditions: 25 °C during a 16/8 h light/dark cycle, light density of 8000 lux. In consideration of rigorous experimental design, original algae cells were purified on solid medium and purified algae cells played the role of seed cells in follow-up experiments. Seed cells were inoculated at 5% (*v*/*v*) into a 2 L Erlenmeyer flask containing 1 L medium and grown to logarithmic phase (about 5 days). The algae cells were collected by centrifuging for 5 min at 5000× *g* at 4 °C and subjected to different stress treatments. For experimental groups, algae cells were transferred to fresh DM medium (containing 1.5 M NaCl) with an additional 3.0 M NaCl (high salt stress), 1.5 M sorbitol (hypertonic stress) and 0.4 mM H_2_O_2_ (oxidative stress), respectively. Algae cells were transferred to fresh normal medium and used as a control group. All samples were cultivated 2 h prior to cell collection, and the collected algae cells were immediately stored in liquid nitrogen for further analysis. Three independent experimental replicates were performed.

### 4.2. Transcriptome Sequencing, Reads Assembly and Functional Annotation

*D. salina* cells under normal growth conditions and stress treatment were sampled for RNA isolation (TRIzol method), and the mRNA with polyA structure in total RNA was enriched by Oligo(dT) magnetic beads, and then the enriched mRNA was broken into 200–300 bp fragments by ion interruption for cDNA library construction. Considering the biological repetition, a total of 12 cDNA libraries were constructed and paired-end (PE) sequencing of these libraries based on the Illumina NextSeq500 sequencing platform was performed. Raw data were first processed to filter low-quality reads (the number of bases with an error rate >1%), reads containing adapters and reads less than 50 bp. After filtration, high-quality sequences (clean data) were assembled into transcript sequences using software Trinity (version r20140717, k-mer 25 bp) and the longest transcripts of each gene were extracted as a representative sequence of the gene, called Unigene. For functional annotation, the unigene sequences were compared against the Nr (NCBI non-redundant protein sequences), Swiss-port (http://web.expasy.org/docs/swiss-prot_guideline.html (accessed on 21 September 2016)), GO (Gene Ontology; http://geneontology.org/ (accessed on 21 September 2016)) [62], KEGG (Kyoto Encyclopedia of Genes and Genomes; http://www.kegg.jp/ (accessed on 21 September 2016)) [63] and EggNOG (evolutionary genealogy of genes: Non-supervised Orthologous Groups; http://eggnog.embl.de/version_4.0.beta/ (accessed on 21 September 2016)) [64] databases to obtain the annotation and categories information of unigenes. GO and KEGG annotations were performed by BLAST2GO (v2.7) [65] software and KAAS (KEGG Automatic Annotation Server; http://www.genome.jp/tools/kaas/ (accessed on 21 September 2016)), respectively. The clusterProfiler 4.0 was used for enrichment analysis [66].

### 4.3. Differential Expression Analysis of Transcripts

The clean reads of each sample were compared to the reference sequences. The quantitative software RSEM (v1.2.31) [67] was used to count the number of reads mapped to each transcript and calculate the Fragments Per Kilobase of exon model per Million mapped fragments (FPKM) value for each gene. Differential expression unigenes were screened by DESeq (version 1.18.0) with a standard |log2 (fold change) | >1 & *p*-value < 0.05.

### 4.4. Intracellular Starch Staining of D. salina

Algae cells in logarithmic phase were treated with high salt stress for 2 h, 4 h, 8 h and 12 h. The cells were collected by centrifugation (5 min at 5000× *g*) and fixed with formaldehyde-acetic acid-ethanol fixative (FAA) solution. After 24 h of fixation, the cells were dehydrated, permeabilized, embedded in paraffin and sectioned. The sections were stained with potassium iodide–iodine solution for 5–10 min and then observed with microscopy. Analysis was performed of the area of starch-stained regions in each cell using ImageJ (https://ij.imjoy.io/).

### 4.5. RT-qPCR Analysis

Eight unigenes were randomly selected from each treatment for RT-qPCR analysis to verify the reliability and reproducibility of the data from RNA-seq. In addition, the expression patterns of 6, 6 and 12 unigenes encoding key enzymes involved in starch hydrolysis and glycerol biosynthesis, antioxidant enzymes and transcription factors, respectively, were monitored by RT-qPCR. These unigenes were selected based on statistically significant expression differences. Primers were designed according to the sequences of selected unigenes using Primer Premier 5, and were synthesized by TsingKe Biotech (Chengdu, China). Algae cells from control and different treatments were collected for RNA isolation using the Trizol Reagent (Invitrogen, Waltham, MA, USA) according to the manufacturer’s instructions. To ensure the rigor of the experiments, equivalent RNA (1 µg) was reverse-transcribed into cDNA using PrimeScript™RT reagent Kit with gDNA Eraser (Perfect Real Time) (Takara, Tokyo, Japan). For RT-qPCR, *beta tubulin* was used as the internal reference gene under high salt stress and *18s rRNA* was used as the internal reference gene under hypertonic stress and oxidative stress and a CFX96 Real-Time PCR Detection System (Bio-Rad, Hercules, CA, USA) was used. Each reaction was carried out in 25 μL of total volume containing 12.5 μL SYBR ^®^ Premix Ex Taq^™^ II (Tli RNaseH Plus) (Takara), 1 μL forward primer (10 μM), 1 μL reverse primer (10 μM), 2 μL cDNA and 8.5 μL ddH_2_O. The reaction conditions were as follows: initial denaturation at 95 °C for 30 sec, followed by 40 cycles at 95 °C for 5 sec and 60 °C for 30 sec. Each sample was set to three repetitions to ensure the credibility of the experimental results. The 2 ^−∆∆Ct^ method [68] was used to analyze the relative expression of genes.

## 5. Conclusions

In this study, we investigated the molecular mechanisms of *D. salina* under salt stress. It was found that *D. salina* adapted to salt stress by significantly adjusting starch and sucrose metabolism and glycerol metabolism pathways. Starch catabolism and glycerol synthesis play key roles in maintaining cellular osmotic pressure homeostasis. Meanwhile, the G3P shuttle mechanism was involved in the regulation of redox homeostasis, helping the cells to cope with the redox imbalance caused by external stress. In addition, an R2R3-MYB transcription factor (c23845_g1) was identified, which was significantly up-regulated and expressed under multiple stress conditions, and may enhance the antioxidant capacity of cells by regulating the transcription of ROS-related genes, thus improving the tolerance of *D. salina* to different stresses. These findings not only enhance our understanding of the molecular mechanisms underlying the adaptation of *D. salina* to salt stress and its pleiotropic effects but also provide important genetic resources and theoretical basis for the future utilization of *D. salina* for biotechnological and agricultural applications.

## Data Availability

The transcriptome data has been uploaded to NCBI under the accession number GSE111682.

## Supplementary Materials

The following supporting information can be downloaded at: https://www.mdpi.com/article/10.3390/ijms26147019/s1.
