# Supplementary material for: Glycerol Biosynthesis Pathways from Starch Endow Dunaliella salina with the Adaptability to Osmotic and Oxidative Effects Caused by Salinity
Source: Int J Mol Sci. 2025 Jul 21;26(14):7019. doi: 10.3390/ijms26147019 (PMC12296084; doi:10.3390/ijms26147019)
Supplement: Supplementary file 1 [file ijms-26-07019-s001.zip › ijms-3678326-supplementary.pdf]

Table S1. Data filtering summary

Sample	Clean Reads No.	Clean Data(bp)	Clean Reads %	Clean Data %
01	40,686,386	6,102,957,900	73.91%	73.91%
02	41,086,298	6,162,944,700	88.25%	88.25%
03	49,323,806	7,398,570,900	83.00%	83.00%
11	47,644,970	7,146,745,500	84.35%	84.35%
12	40,906,466	6,135,969,900	84.25%	84.25%
13	40,856,548	6,128,482,200	89.75%	89.75%
21	40,652,874	6,097,931,100	84.97%	84.97%
22	40,578,960	6,086,844,000	83.44%	83.44%
23	41,593,886	6,239,082,900	84.30%	84.30%
31	40,823,050	6,123,457,500	82.07%	82.07%
32	40,311,704	6,046,755,600	85.82%	85.82%
33	43,784,114	6,567,617,100	88.06%	88.06%

01, 02, 03: control, 11, 12, 13: high salt stress, 21, 22, 23: oxidative stress, and 31, 32, 33: hypertonic stress.

Table S2. Summary of assembly results

	Contig	Transcript	Unigene
Sequence Number	74,448,922	101,730,718	51,966,016
Max. Length (bp)	218,608	126,222	82,333
Mean Length (bp)	17,240	17,147	17,147
N50 (bp)	340.56	805.97	631.17
N50 Sequence No.	497	1,337	1,036
N90 (bp)	30,254	22,147	12,777
N90 Sequence No.	144	308	253
GC%	155,929	84,645	59,182

Table S3. Summary of expression difference analysis

Case	Control	Up-regulated Unigene		Down-regulated Unigene		Total DE Unigene	
		Number	%	Number	%	Number	%
G0	G1	2964	3.6	1756	2.13	4720	5.73
G0	G2	1011	1.23	100	0.12	1111	1.35
G0	G3	2228	2.71	383	0.47	2611	3.17
G1	G2	2004	2.43	2584	3.14	4588	5.57
G1	G3	1604	1.95	1091	1.33	2695	3.27
G2	G3	1080	1.31	497	0.6	1577	1.92

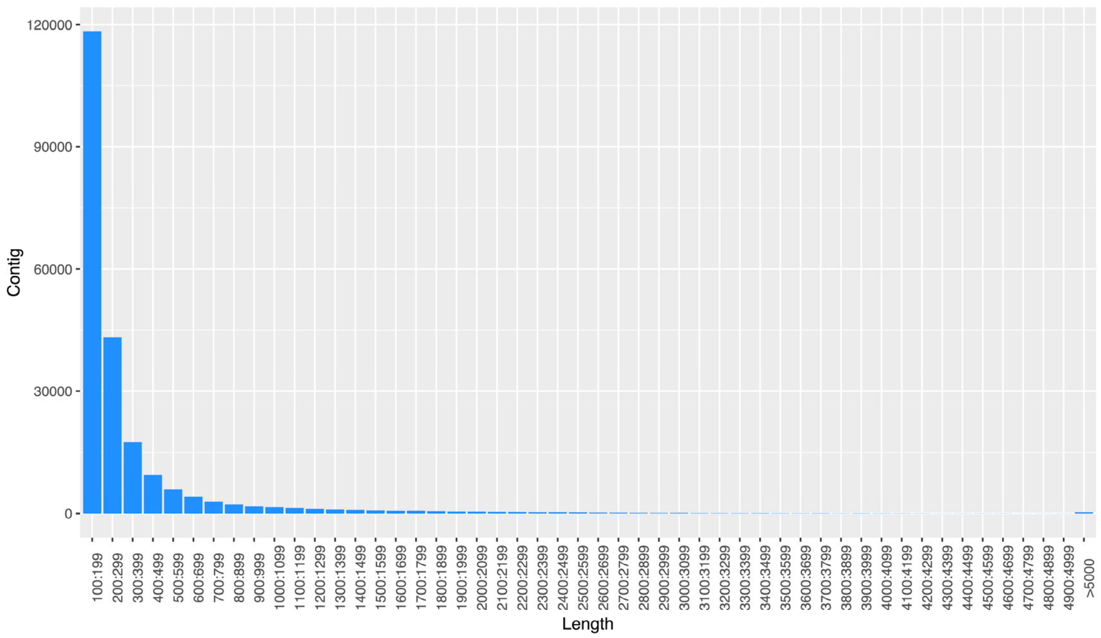

Figure S1. Transcript Length Frequency Distribution. The horizontal coordinate indicates the length interval of the sequence, and the vertical coordinate indicates the number of Transcript sequences with lengths within that interval. Generally the number of sequences decreases as the length increases.

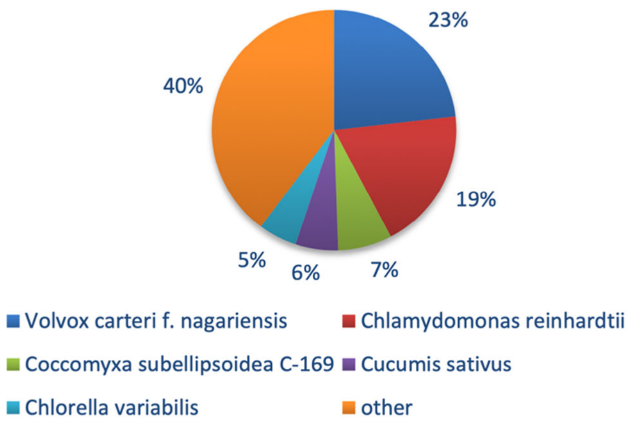

Figure S2. NR blast species distribution. BLAST searches of *D. salina* unigenes against the NR database identified homologous sequences across diverse species.

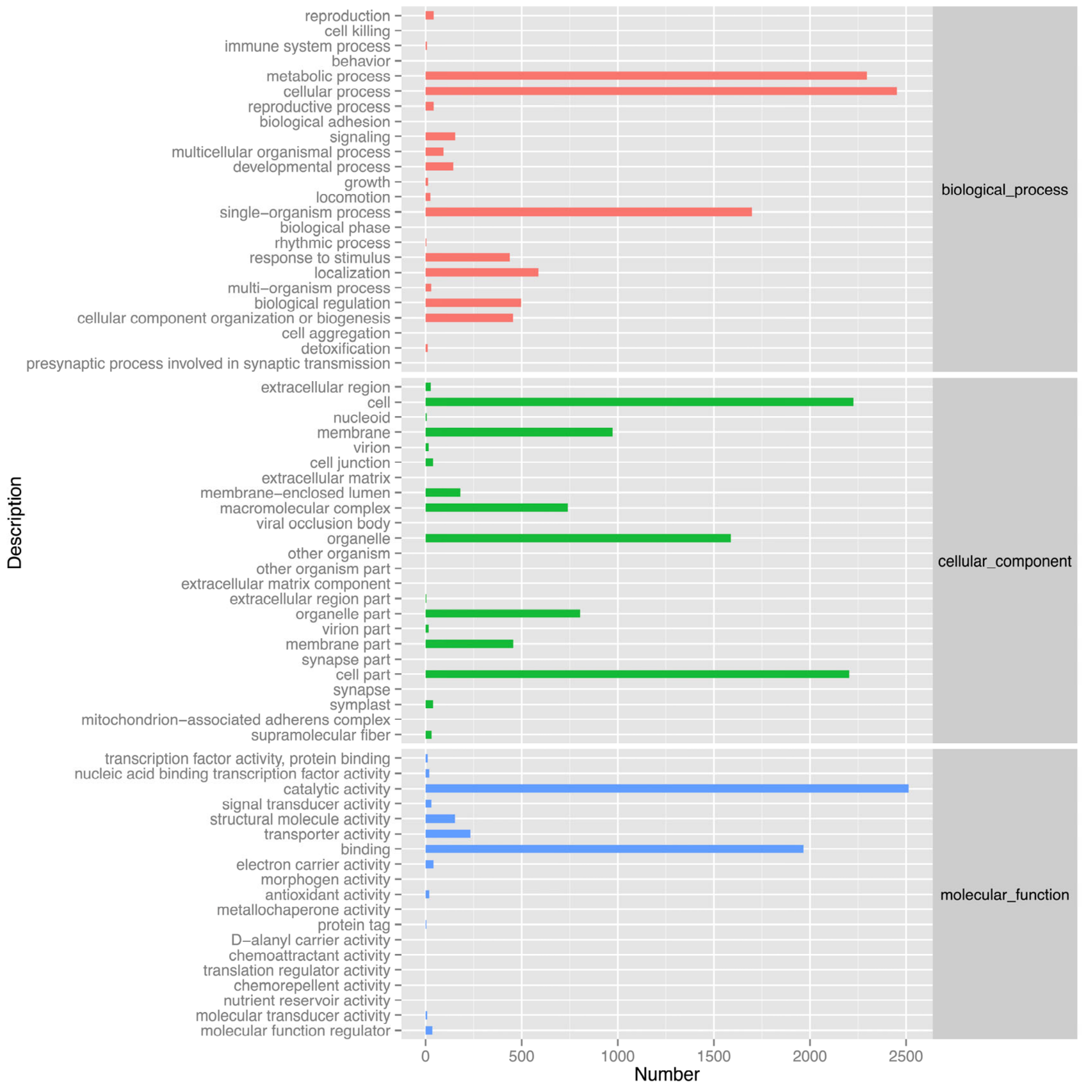

Figure S3. GO Classification Annotation. The vertical coordinate represents the GO term and the horizontal coordinate represents the number of Unigenes annotated to that GO term.

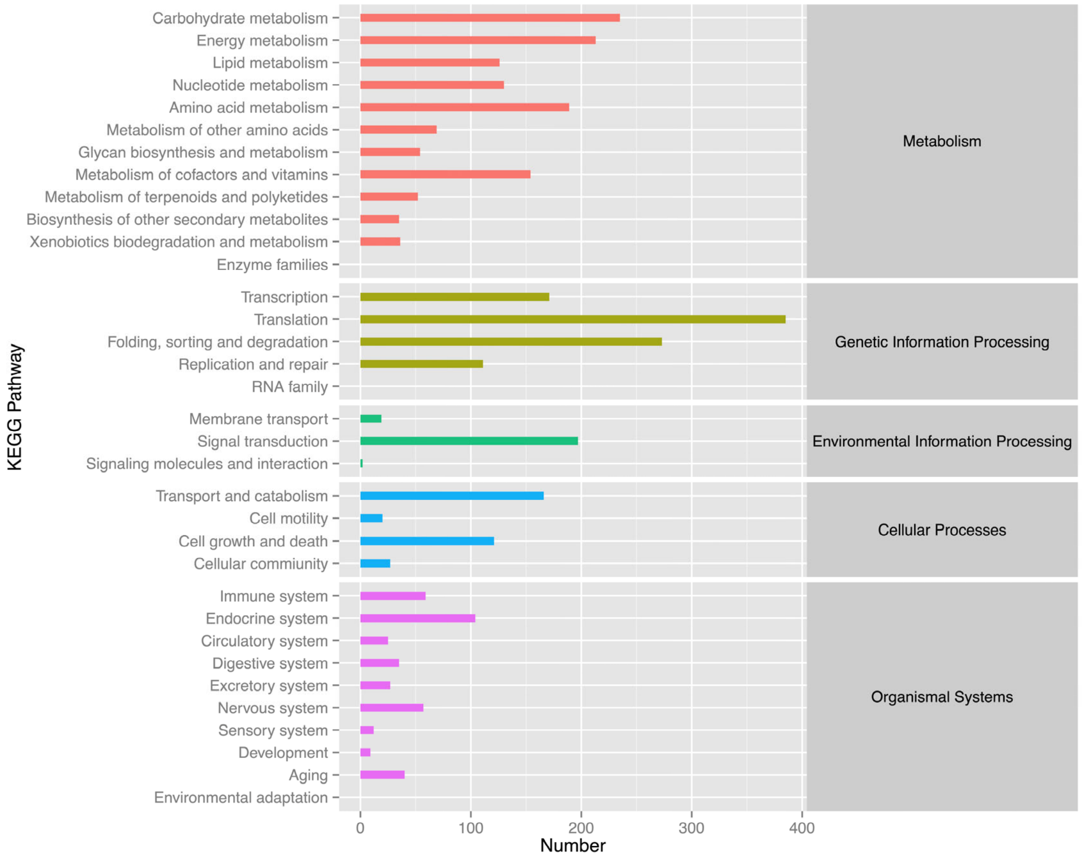

Figure S4. KEGG Annotation. The vertical coordinate represents the KEGG term and the horizontal coordinate represents the number of Unigenes annotated to that KEGG term.
